# Supplementary material for: Investigation of oral macrolide prescriptions in Japan using a retrospective claims database, 2013–2018
Source: PLoS One. 2023 Jun 22;18(6):e0287297. doi: 10.1371/journal.pone.0287297 (PMC10286965; doi:10.1371/journal.pone.0287297)
Supplement: Attachment — Submitted filename: review1.docx [file pone.0287297.s001.docx]

**Review Report**

**Investigation of oral macrolide prescriptions in Japan using a retrospective claims database, 2013–2018**

The manuscript is a retrospective observational study conducted using data extracted from Japan

Medical Database Claim from January 2013 to December 2018. The proportion of all oral antimicrobials

and macrolides used during this period and the diseases for which macrolides were used in the three

years before and after the Antimicrobial Resistance Action Plan were determined separately for acute and chronic diseases. The number of prescriptions for macrolides constituted approximately 30% of those for all oral antimicrobials; of these, clarithromycin accounted for approximately 60%. Most prescriptions for acute diseases were for common cold, whereas for chronic diseases were allergic and dermatological disorders. The pattern of prescription did not change before and after the AMR Action Plan. The authors clinched that these results indicated that appropriate macrolide use involves a review of their use for common cold along with appropriate evaluation of their long term use for skin and allergic diseases. They also concluded the need for further fact-finding studies and more AMR measures.

**Comments:**

1. The manuscript is poorly written. It requires extensive editing, Grammar correction, and rephrasing.
2. References are placed beside sentences in a confusing way.
3. The structure of some sentences is contradictory, example paragraph 290- 295.
4. Abbreviations appear without explanation. Their explanation then appear after 2 or three pages; example JMDC in page 3.
5. Explanation of Japan Medical Database Claim (JMDC), the core of the study, should be placed in the introduction; what it is, importance, data contained and comparison with similar databases of other countries. It is not appropriate to be placed in the study design.
6. DDD, and DID

- Reference [12] does not define DID; it defines DDD.
- DID is not a measure of the number of people. Lines 83 and 84 need to be rephrased. DDD and DID should be explained in a clearer way with proper placement of references.
- DDDs are not established for all medicines with an ATC code. Major drug groups without DDDs are, in addition to others, ophthalmologicals and otologicals. Table 1 showing that among characteristics of patients and facilities where macrolides were prescribed are otolaryngology hospitals which are expected to provide treatment for patients with ontological problems. For such patients DDDs are not the right choice. Table 2 shows infections for which macrolides were prescribed and including conjunctivitis.
- Page 10, patients were categorized into 10 age group (0-3, 4-6, 7-12………………) e.g., which are considered children groups. It is to be emphasized that DDD for children is challenging. Assessing frequency of drug use in children is not possible by using DDDs due to the changeability of children’s doses, according to WHO reference <https://www.who.int/tools/atc-ddd-toolkit/about-ddd>

7. **Statistical analysis:** statistical analysis of pattern of macrolide prescription which was not done in the current work was necessary in comparison with standard treatment regimens. Such analysis would identify the prescription behaviors of clinicians and could determine rational and rational use of macrolides. It would also show cases where antibiotics should not be prescribed. Such findings would form the objectives for designing educational programs to raise awareness among prescribers, so fulfilling recommendations of the country action plan.
8. **Results**

In order to add more power to the study, it is recommended to translate your findings into financial cost. This can be done by determining the cost- benefit of eliminating unnecessary use of macrolides. In addition to be clearer, this will help get the message across to prescribing clinicians. These findings could be presented during orientation programs to enhance prudent use of macrolides.

Another important point is to highlight disease where macrolides were to be prescribed, but they were not. It is important to decrease the frequency of macrolides use, and at the same time do not interfere with providing proper healthcare service.

9. **Discussion**

- What is the reason that clarithromycin was the most commonly prescribed?? Is due to price factor or more activity of drug companies?
- Line 297: what are the current AMR measurses????
- Line 298: is there any rapid test done to exclude bacterial infection in cases of common cold as CRP for example. What do you do if a neutropenic patient is coming with common cold. Clear guidelines should be provided for prescribing macrolides in specific situations.
- As regards long term use, what do you make to minimize emergence of resistance
- Authors listed limitation of the study. The second limitation is a serious one. The question is what is the relation of this study with medicine??? A professional statistician can make similar work. Authors mentioned in lines 361, 362 that their shortcomings were also pointed out in previous studies, so why they did not have different approach. Since there is no data base in Japan with sufficiently verified diagnosis and clinical information, such studies are of no value
- Subsequent limitation starting at line 364, destroys the study design of using DDDs. A finding that is already established since DDD is sometimes a dose that is rarely or never prescribed because it is an average of two or more commonly used doses.
- The fourth limitation (line 71) is again falling in the same mistake of other authors.
- Line 382, a serious concept in providing high quality patient care is the term (decrease prescription), the right term is to (rationalize prescription).

**Conclusions:**

1. **I do not recommend publishing this article due to all I have listed before. The work has poor relation to medicine.**
2. **The study could be a part of more meaningful work. It could be the first portion of an interventional study at less wide scale.**
