# Supplementary material for: Investigation of oral macrolide prescriptions in Japan using a retrospective claims database, 2013–2018
Source: PLoS One. 2023 Jun 22;18(6):e0287297. doi: 10.1371/journal.pone.0287297 (PMC10286965; doi:10.1371/journal.pone.0287297)
Supplement: Attachment — Submitted filename: response_to_reviewer_comments_PlosOne.docx [file pone.0287297.s002.docx]

Feb 15th, 2023

Iddya Karunasagar

*PLOS ONE*

Dear Editor:

We wish to re-submit a manuscript for publication in *PLOS ONE*, titled “**Investigation of oral macrolide prescriptions in Japan using a retrospective claims database, 2013–2018**”. The original journal-supplied reference number for this submission is PONE-D-22-29639.

We have attempted to address the helpful comments of the reviewers and believe that the manuscript is now much improved. Below, we provide a point-by-point response to the reviewers’ comments. Major changes are highlighted by red text in the revised manuscript.

This manuscript has not been published or presented elsewhere in part or in entirety and is not under consideration by another journal. We have read and understood your journal’s policies, and we believe that neither the manuscript nor the study violates any of these. There are no conflicts of interest to declare.

Thank you for your consideration. I look forward to hearing from you.

Sincerely,

Satoshi Ide, MD

Assistant Professor

Department of Infection Control and Prevention,

Tokyo Medical and Dental University Hospital

[babytiger0313@gmail.com](mailto:babytiger0313@gmail.com)

**Response to Reviewer #1**

[Comment 1] Trend analysis statements are not clearly supported by statistical analysis. Need to give details of how the trend analysis was performed. Comparing 3 years before and after may not be statistically sound.

**Response:** Thank you for your suggestion. We performed a Cochran–Armitage trend test for statistical evaluation (Figure 1-B and Figure 2) and have revised the results section (p12-13 l188-194) as follows:

“The proportion of prescriptions for cephalosporins and macrolides was significantly decreased (p<0.001), whereas that for quinolones and penicillin was significantly increased (p<0.001). The proportions of types of prescribed macrolides by year from 2013 to 2018 are also shown in Figure 2. Clarithromycin accounted for the largest proportion (more than 60%) in all years. The proportion of prescriptions for clarithromycin was significantly decreased (p<0.001), whereas that for azithromycin, roxithromycin, and erythromycin was significantly increased (p<0.001).”

For detailed evaluation of changes in prescriptions of macrolides before and after the Action Plan, we needed to evaluate the proportion of macrolides prescribed to a population with diagnosis of a certain disease; however, this is not evaluable from this database because the denominators of Table 2 and Table 3 are number of cases prescribed macrolides.

**Response to Reviewer #2**

[Comment 1] The manuscript is poorly written. It requires extensive editing, Grammar correction, and rephrasing.

**Response:** We have thoroughly reviewed the manuscript to eliminate grammatical mistakes.

[Comment 2] References are placed beside sentences in a confusing way.

**Response:** Thank you for your observation. We have moved references in the text to the ends of the sentences.

3. The structure of some sentences is contradictory, example paragraph 290- 295.

**Response:** Thank you for your comment. We have revised the sentences (P24-25, l285-298) as follows:

“Our study shows a decrease in the proportion of macrolide prescriptions from 2013 to 2018 (Figure 1B), although the overall number of cases increased each year (Figure 1A). Clarithromycin showed a decreasing trend, while azithromycin, roxithromycin, and erythromycin showed increasing trends (Figure 2). The 10 most common acute diseases for which macrolides were prescribed showed no change before and after the Action Plan. National antimicrobial sales in Japan have been reported to have decreased since 2016 [26]. Therefore, it was speculated that the decrease in overall antimicrobial use of antimicrobials for cold syndromes and other conditions due to the AMR measures may have influenced the decrease in the percentage of macrolide antimicrobials used. The reasons clarithromycin is frequently used in Japan is likely because it is a broad-spectrum antibacterial agent with few side-effects, acts for a long time in the body, and is expected to have an immunomodulating effect. Further investigation is needed to clarify the factors that contributed to the decrease in the use of macrolide antibacterial agents [11].

”

4. Abbreviations appear without explanation. Their explanation then appear after 2 or three pages; example JMDC in page 3.

**Response:** Thank you for pointing this out. Before 2018, the JMDC was called the Japan Medical Database Claim. However, today, JMDC is the official name of the company. Therefore, we would this description to remain unchanged.

5. Explanation of Japan Medical Database Claim (JMDC), the core of the study, should be placed in the introduction; what it is, importance, data contained and comparison with similar databases of other countries. It is not appropriate to be placed in the study design.

**Response:** Thank you for your comment. Other papers using JMDC data have also included details about JMDC in the study design; we feel that this is also appropriate for our study. However, we have also added some information about the database in the Introduction (p7 l97-100).

6. DDD, and DID

-Reference [12] does not define DID; it defines DDD.

-DID is not a measure of the number of people. Lines 83 and 84 need to be rephrased. DDD and DID should be explained in a clearer way with proper placement of references.

**Response:** Thank you for your comment. We have fixed the citations and revised the text (p6 l77-79) as follows:

“The defined daily dose (DDD) is the assumed average maintenance dose per day for a drug used for its main indication in adults. The DDD/1000 inhabitants/day (DID) is a measure of the number of people and is used to correct for differences in the usage of different antimicrobials in different populations.”

-DDDs are not established for all medicines with an ATC code. Major drug groups without DDDs are, in addition to others, ophthalmologicals and otologicals. Table 1 showing that among characteristics of patients and facilities where macrolides were prescribed are otolaryngology hospitals which are expected to provide treatment for patients with ontological problems. For such patients DDDs are not the right choice. Table 2 shows infections for which macrolides were prescribed and including conjunctivitis.

-Page 10, patients were categorized into 10 age group (0-3, 4-6, 7-12………………) e.g., which are considered children groups. It is to be emphasized that DDD for children is challenging. Assessing frequency of drug use in children is not possible by using DDDs due to the changeability of children’s doses, according to WHO reference <https://www.who.int/tools/atc-ddd-toolkit/about-ddd>

**Response:** Thank you for your comment. We understand that DDDs have several limitations. However, the evaluation indicator in this study was the number of cases, not DDD. Therefore, we do not think that additional explanations of DDD are necessary.

**7. Statistical analysis:** statistical analysis of pattern of macrolide prescription which was not done in the current work was necessary in comparison with standard treatment regimens. Such analysis would identify the prescription behaviors of clinicians and could determine rational and rational use of macrolides. It would also show cases where antibiotics should not be prescribed. Such findings would form the objectives for designing educational programs to raise awareness among prescribers, so fulfilling recommendations of the country action plan.

**Response:** Thank you for your comment. We could not evaluate the appropriateness of the disease name given that this is a retrospective database, and the data do not include groups of patients who were not prescribed macrolides. Moreover, it is difficult to define a standard macrolide treatment for some diseases (e.g., allergic or dermatologic diseases) because various studies and guidelines differ in their judgment of the appropriateness of macrolides. Therefore, we decided not to perform a statistical analysis to compare the data with the standard treatment.

**8. Results**

In order to add more power to the study, it is recommended to translate your findings into financial cost. This can be done by determining the cost- benefit of eliminating unnecessary use of macrolides. In addition to be clearer, this will help get the message across to prescribing clinicians. These findings could be presented during orientation programs to enhance prudent use of macrolides.

Another important point is to highlight disease where macrolides were to be prescribed, but they were not. It is important to decrease the frequency of macrolides use, and at the same time do not interfere with providing proper healthcare service.

**Response:** We agree with your suggestions. However, the database does not include the financial costs or diseases for which macrolides were originally used. We would like to explore this issue in future research.

9. **Discussion**

-What is the reason that clarithromycin was the most commonly prescribed?? Is due to price factor or more activity of drug companies?

**Response:** Thank you for your comment. We have added the following sentences (p25 l294-298) as follows:

“The reasons clarithromycin is frequently used in Japan is likely because it is a broad-spectrum antibacterial agent with few side-effects, acts for a long time in the body, and is expected to have an immunomodulating effect. Further investigation is needed to clarify the factors that contributed to the decrease in the use of macrolide antibacterial agents [11]”

-Line 297: what are the current AMR measurses????

**Response:** Thank you for your comment. We have added the following sentences (p25 l299-302) as follows:

“Current AMR measures include antimicrobial awareness surveys, surveillance of antimicrobial use, and the development of guidance and educational tools around the proper use of antimicrobials for primary care physicians [27]. However, there is still no approach targeting reduction of macrolides.”

-Line 298: is there any rapid test done to exclude bacterial infection in cases of common cold as CRP for example. What do you do if a neutropenic patient is coming with common cold. Clear guidelines should be provided for prescribing macrolides in specific situations.

**Response:** Thank you for your comment. We apologize for the inadequate explanation. Since the database does not include information on the results of blood tests, or the reason for diagnosis, we are not able to address this issue.

-As regards long term use, what do you make to minimize emergence of resistance

**Response:** Thank you for your comment. We have added the following sentence (p27 l337-340) as follows:

“To minimize emergence of resistance, the evidence for diseases requiring low-dose long-term administration should be reaffirmed, and physicians should be educated avoid prescribing for prolonged cough only.”

-Authors listed limitation of the study. The second limitation is a serious one. The question is what is the relation of this study with medicine??? A professional statistician can make similar work. Authors mentioned in lines 361, 362 that their shortcomings were also pointed out in previous studies, so why they did not have different approach. Since there is no data base in Japan with sufficiently verified diagnosis and clinical information, such studies are of no value.

**Response:** Thank you for your comment. Although previous studies have shown that oral macrolides are used more than other antimicrobials, there have been no studies that clarified their usage. There may be a novelty that we analyzed name of diseases and the analysis was divided into acute and chronic phases. Although the lack of a database containing clinical information in Japan is an important issue, we believe that it is necessary to first identify trends using retrospective big data for the rationalization of use of macrolides in the future.

-Subsequent limitation starting at line 364, destroys the study design of using DDDs. A finding that is already established since DDD is sometimes a dose that is rarely or never prescribed because it is an average of two or more commonly used doses.

-The fourth limitation (line 71) is again falling in the same mistake of other authors.

**Response:** Thank you for your comment. The evaluation indicator in this study was the number of cases, not DDD.

-Line 382, a serious concept in providing high quality patient care is the term (decrease prescription), the right term is to (rationalize prescription).

**Response:** Thank you for your comment. We have revised the text as suggested.

**Reviewer #3:**

#1. p9 l135 J01FA12 corresponds to rokitamycin instead of roxithromycin.

**Response:** Thank you for your comment. We have corrected this misprint.

#2. Results section contain many errors. The authors quote number of prescirptions as well as proportion and some mix-up occurs. E.g. p12, l187, they mention the number of prescriptions for cephalosporins and penicillins show a decreasing trend ... but in figure 1-A it is definitely increasing!
**Response:** Thank you for your comment. We have corrected this sentences (p12 l188-190) as follows:

“The proportion of prescriptions for cephalosporins and macrolides was significantly decreased (p<0.001), whereas that for quinolones and penicillin was significantly increased (p<0.001)..”

#3. The section on "Epidemiology of diseases for which macrolides were prescribed in the three years before and after the Action Plan" is not well written. It starts with "Table 2 shows the top 10 most prescribed macrolides for acute illnesses in 2013–2015 (n = 13,555,601) and 2016–2018 (n = 23,684,663). Clarithromycin was prescribed for nine of the 10 top most diseases, and azithromycin was prescribed for one disease. " Firstly, the table shows the 10 most common acute diseases for which macrolides were prescribed and not 10 most prescribed macrolides. Secondly, the authors mean that clarithromycin was THE MOST COMMONLY prescribed macrolide for 9 of the 10 diseases on the list.

**Response:** Thank you for your comment. We have corrected the sentences (p17-18, l243-246 and l250-253) as follows:

“Table 2 shows the 10 most common acute diseases for which macrolides were prescribed in 2013–2015 (n = 13,555,601) and 2016–2018 (n = 23,684,663). Clarithromycin was the most commonly prescribed macrolide for 9 of the 10 diseases on the list.”

“A similar analysis was performed, and the 10 most common chronic diseases for which macrolides were prescribed in 2013–2015 (n = 1,793,965) and 2016–2018 (n = 3,660,871) [Table 3]. Clarithromycin was also the most commonly prescribed macrolide for 9 of the 10 diseases, and roxithromycin was prescribed for 1 disease.”

#4. Table 2 itself is presented as "List of acute infectious diseases for which macrolides were prescribed for less than 14 days" but includes non-infectious diseases such as asthma and allergic rhinitis.
**Response:** Thank you for your comment. We have corrected the titles to “List of acute infectious diseases for which macrolides were prescribed for less than 14 days” (Table 2) and “List of chronic infectious diseases for which macrolides were prescribed for more than 14 days” (Table 3).

#5. p14, l229 "Comparisons between 2013–2015 and 2016–2018 showed a decrease in the age groups 0–3, 4–6, and 7–12 years from 8.1%, 6.6%, and 8.7% to 6.5%, 5.4%, and 8.0%, respectively." It is the PROPORTION OF PRESCRIPTIONS which occurred in these age groups which decreased.
**Response:** Thank you for your comment. We have revised the sentence (p14 l228-230) as follows:

“Comparisons of the proportion of prescriptions between 2013–2015 and 2016–2018 showed a decrease in the 0–3, 4–6, and 7–12 years age groups from 8.1%, 6.6%, and 8.7% to 6.5%, 5.4%, and 8.0%, respectively.”

#6. In the discussion section, lines 290-292, "Our study revealed a downward trend in macrolide prescription among all antimicrobials from 2013–2018,..." but the number actually doubled as seen in figure 1A.
**Response:** Thank you for your comment. We apologize for the inadequate explanation. Figure 1A shows number of cases; owing to an increase in the number of registrations to JMDC, the total number of cases approximately doubled from 2013 to 2018. Therefore, comparison was made by percentage (Figure 1B), which showed a downward trend. To avoid misunderstanding, we have revised the text (p24 l285-288) as follows:

“Our study shows a decrease in the proportion of macrolide prescriptions from 2013 to 2018 (Figure 1B), although the overall number of cases increased each year (Figure 1A). Clarithromycin showed a decreasing trend, while azithromycin, roxithromycin, and erythromycin showed increasing trends (Figure 2).”
