# Supplementary material for: Investigation of oral macrolide prescriptions in Japan using a retrospective claims database, 2013–2018
Source: PLoS One. 2023 Jun 22;18(6):e0287297. doi: 10.1371/journal.pone.0287297 (PMC10286965; doi:10.1371/journal.pone.0287297)
Supplement: Attachment — Submitted filename: Response to Reviewers.docx [file pone.0287297.s003.docx]

May 13th, 2023

Iddya Karunasagar

*PLOS ONE*

Dear Editor:

We wish to re-submit a manuscript for publication in *PLOS ONE*, titled “**Investigation of oral macrolide prescriptions in Japan using a retrospective claims database, 2013–2018**”. The original journal-supplied reference number for this submission is PONE-D-22-29639.

We have attempted to address the helpful comments of the reviewers and believe that the manuscript is now much improved. Below, we provide a point-by-point response to the reviewer’s comments. The changes are highlighted by red text in the revised manuscript.

This manuscript has not been published or presented elsewhere in part or in entirety and is not under consideration by another journal. We have read and understood your journal’s policies, and we believe that neither the manuscript nor the study violates any of these. There are no conflicts of interest to declare.

Thank you for your consideration. I look forward to hearing from you.

Sincerely,

Satoshi Ide, MD

Assistant Professor

Department of Infection Control and Prevention,

Tokyo Medical and Dental University Hospital

[babytiger0313@gmail.com](mailto:babytiger0313@gmail.com)

**Response to Reviewer #3**

[Comment 1] Lines 55-57: data refer to yearly number of deaths. “yearly” should be added. “By 2050, if no action is taken against AMR, it is expected to cause 10 million deaths YEARLY worldwide, which exceeds the number of deaths caused by cancer [4, 5]”
**Response:** Thank you for your comment. We added the word “yearly” in between the sentence.

[Comment 2] Lines 94-100: the same sentence is repeated twice!

**Response:** Thank you for your comment. We have deleted the overlapped sentence.

[Comment 3] Line 159: L.pneumophila instead of L. pneumoniae

**Response:** Thank you for pointing out our mistake. We corrected a typo.

[Comment 4] Line 253: and roxithromycin was MOST COMMONLY prescribed for 1 disease

**Response:** Thank you for your comment. We added the word “most commonly” in between the sentence.

[Comment 5] Line 258: Should this not be “2016-2918” instead of “2018”?

**Response:** Thank you for pointing out this point. We added “2016-“ in front of 2018 to make it correct sentence.

[Comment 6] Line 269: Table 3. The word “Infectious” should be removed.

**Response:** Thank you for your comment. We have deleted the word “infectious”.
